# Excito-Secretory System of Nerves

**Published:** 1857-08

**Authors:** 


*Excito-Secretory System of Nerves.*—Dr. Marshall Hall of London has yielded the credit of discovery of the excitory secretory system of nerves to Dr. H. F. Campbell, of Augusta, Georgia, recently elected Professor of Anatomy in the Medical College of Georgia.

